# In-target production of [^11^C]CH_4_ from a nitrogen/hydrogen gas target as a function of beam current, irradiation time, and target temperature

**DOI:** 10.1186/s41181-024-00255-1

**Published:** 2024-03-25

**Authors:** Semi Helin, Johan Rajander, Jussi Aromaa, Eveliina Arponen, Jatta S. Helin, Olof Solin

**Affiliations:** 1 Turku PET Centre, Radiopharmaceutical Chemistry Laboratory, University of Turku, Kiinamyllynkatu 4-8, 20520 Turku, Finland; 2 Turku PET Centre, Accelerator Laboratory, Åbo Akademi University, Kiinamyllynkatu 4-8, 20520 Turku, Finland; 3 MediCity Research Laboratory, University of Turku, Tykistökatu 6 A, 20520 Turku, Finland; 4 Turku PET Centre, Preclinical Imaging Laboratory, University of Turku, Tykistökatu 6 A, 20520 Turku, Finland; 5 Department of Chemistry, University of Turku, Henrikinkatu 2, 20500 Turku, Finland; 6 Turku PET Centre, Turku University Hospital, Kiinamyllynkatu 4-8, 20520 Turku, Finland

**Keywords:** Carbon-11, [^11^C]methane, Targetry, PET, Haber–Bosch

## Abstract

**Background:**

Production of [^11^C]CH_4_ from gas targets is notorious for weak performance with respect to yield, especially when using high beam currents. Post-target conversion of [^11^C]CO_2_ to [^11^C]CH_4_ is a widely used roundabout method in ^11^C-radiochemistry, but the added complexity increase the challenge to control carrier carbon. Thus in-target-produced [^11^C]CH_4_ is superior with respect to molar activity. We studied the in-target production of [^11^C]CO_2_ and [^11^C]CH_4_ from nitrogen gas targets as a function of beam current, irradiation time, and target temperature.

**Results:**

[^11^C]CO_2_ production was practically unchanged across the range of varied parameters, but the [^11^C]CH_4_ yield, presented in terms of saturation yield Y_SAT_(^11^CH_4_), had a negative correlation with beam current and a positive correlation with target chamber temperature. A formulated model equation indicates behavior where the [^11^C]CH_4_ formation follows a parabolic graph as a function of beam current. The negative square term, i.e., the yield loss, is postulated to arise from Haber–Bosch-like NH_3_ formation: N_2_ + 3H_2_ → 2NH_3_. The studied conditions suggest that the NH_3_ (liq.) would be condensed on the target chamber walls, thus depleting the hydrogen reserve needed for the conversion of nascent ^11^C to [^11^C]CH_4_.

**Conclusions:**

[^11^C]CH_4_ production can be improved by increasing the target chamber temperature, which is presented in a mathematical formula. Our observations have implications for targetry design (geometry, gas volume and composition, pressure) and irradiation conditions, providing specific knowledge to enhance [^11^C]CH_4_ production at high beam currents. Increased [^11^C]CH_4_ radioactivity is an obvious benefit in radiosynthesis in terms of product yield and molar radioactivity.

**Supplementary Information:**

The online version contains supplementary material available at 10.1186/s41181-024-00255-1.

## Background

[^11^C]CH_4_ has become the preferred cyclotron-produced precursor over [^11^C]CO_2_ for labeling ^11^C-containing radiopharmaceuticals at high molar radioactivity (Noguchi and Suzuki 2003; Andersson et al. 2009). High amounts of [^11^C]CO_2_ can be robustly achieved, but the reliable production of [^11^C]CH_4_ in high yields is problematic using the same ^14^N(p,α)^11^C nuclear reaction for the initial ^11^C generation. Indeed, even though in a 5% H_2_ 95% N_2_ target, the radiolytic reduction of the initial product [^11^C]CN into [^11^C]CH_4_ is 95%–100% at proton beam intensities > 1 eV molecule^−1^ s^−1^ (Ferrieri and Wolf 1983), the yields of harvested [^11^C]CH_4_ in the large scale production fall short of the theoretical ^11^C yield.

For decades, several groups have studied the processes and parameters in gas targets involved in the production of ^11^C and subsequently [^11^C]CH_4_. Recently, the TRIUMF group has been including detailed overviews in their publications (Jahangiri et al. 2016; Uittenbosch et al. 2018), tracing research activities back to the 1960s and the foundational works of H.J. Ache and A.P. Wolf (Ache and Wolf 1966, 1968) on ^11^C hot-atom chemistry in nitrogen gas systems. Ache and Wolf also examined the recoil ^11^C interaction with the target chamber wall, concluding that wall retention is a function of gas pressure and fairly independent from irradiation dose or intensity (Ache and Wolf 1966).

The noted yield declines are mainly attributed to a wall effect (Andersson et al. 2009; Uittenbosch et al. 2018; Buckley et al. 2000, 2004; Koziorowski et al. 2010; Gillings et al. 2012; Zacchia et al. 2018). The contribution of target chamber material, size, and geometry (Buckley et al. 2000, 2004; Koziorowski et al. 2010) to [^11^C]CH_4_ yield also has been studied. Recent discussion (Uittenbosch et al. 2018) has reverted to the role of geometry and dimensions regarding whether the nascent ^11^C reaches the wall before reacting into [^11^C]CH_4_. Uittenbosch et al. explored the disrupting effect of forced target gas circulation on wall retention and described chamber geometry (cylindrical favored over conical) as having a stronger influence on recovery yields than the forced gas circulation. Forced convection still had some effect, disturbing wall retention especially in a conical target chamber. Expanding the earlier studies, focusing mainly on atomic ^11^C, Zacchia et al. also included adsorption of the intermediates and final species in the model of chemical kinetics in ^11^C gas targets (Zacchia et al. 2019). In addition to these factors, we introduced to the targetry community evidence of the strong dependence of [^11^C]CH_4_ recovery yield on target and target chamber temperature (Helin et al. 2010), which others have confirmed (Gillings et al. 2012; Jahangiri et al. 2016).

Despite these investigations, the underlying processes and chemical form of adhering species remain unresolved. [^11^C]CH_4_ production remains poorly understood and is known to be sensitive to the deployed conditions. Consequently, Andersson et al. described the common practice of using an experimentally identified optimal irradiation time for a chosen beam current (Andersson et al. 2009). Equations and models derived and developed from empirical data have proven useful for uncovering common factors and correlations. Examples include temperature distribution (Heselius et al. 1982a), beam penetration (Heselius and Solin 1986; Heselius et al. 1987), pressure rise as a function of beam current (Wojciechowski et al. 1988), density reduction in the beam strike volume (Heselius et al. 1984; Köble et al. 1989; Hällsten and Solin 2002), and yield limiting factors as a function of irradiation time (Buckley et al. 2004). More recently, models have been developed from a theoretical starting point and used to calculate heat transfer coefficients for various target gas and chamber systems (Jahangiri et al. 2016). Furthermore, coupled computational models have been used to optimize target chamber design as it relates to reaction rate density distribution (Peeples et al. 2017).

In the energetic conditions of proton irradiation, various side reactions between the target gas components nitrogen and hydrogen also are anticipated. One of the most obvious would be nitrogen fixation, resembling the famous Haber–Bosch process: N_2_ + 3H_2_ → 2NH_3_ (Briney 2021; Rouwenhorst et al. 2020). Ammonia formation has already been considered (Buckley et al. 2000), this work concluded that the detected NH_3_ quantities in the irradiated gas or the correlation of NH_3_ formation with irradiation time could not explain the declining [^11^C]CH_4_ yields.

Our study of [^11^C]CO_2_ and [^11^C]CH_4_ formation at varied targetry conditions provides systematic and extensive data to support investigation of the involved hot-atom processes and [^11^C]CH_4_ yield suppression. Using our data, known dependencies, and simple mathematical formulae, we present a semi-theoretical model that expresses the current temperature dependency and accurately predicts the yield of [^11^C]CH_4_ at given conditions. We also propose an ammonia phase-state mechanism for the temperature-dependent wall effect.

## Methods

### Instrumentation and materials

#### Cyclotron

All irradiations were carried out using a CC18/9 cyclotron (Efremov Institute of Electrophysical Apparatus, Saint Petersburg, Russia) at the Åbo Akademi Accelerator Laboratory of the Turku PET Centre in Finland. Protons with an energy of 17.0 ± 0.1 MeV (Avila-Rodriguez et al. 2009), attenuated by 0.4 MeV after passage through the inlet foil, were used for all irradiations. Beam currents on the target chamber were up to 40 µA. All irradiations were carried out at an external irradiation position about 5 m downstream of the beam transport line. Two quadrupole lens pairs, as well as a beam-sweeping magnet on this line, ensured uniform particle beam quality for irradiations that were spaced in time across more than a year.

Data on beam current on the target chamber and collimator, target pressure, target chamber temperature, radioactivity readings at several points, and numerous cyclotron-related parameters were stored at a rate of 1 Hz. These data were used to determine average irradiation time, beam current, and target chamber temperature during each irradiation. In all calculations and figures, the measured beam current (nominal current) on the target chamber was corrected by a grid transparency factor to obtain the actual beam current impinging on the target gas.

The expected generation of ^11^C from the ^14^N(p,α)^11^C nuclear reaction was calculated using the well-known yield equation:

1 $$A_{{EOB}} \left( {{}_{{\text{~}}}^{{11}} C} \right) = Y_{{SAT}} \left( {{}_{{\text{~}}}^{{11}} C} \right) \cdot \left( {1 - e^{{ - ln2 \cdot t/T_{{1/2}} }} } \right) \cdot I$$

where A_EOB_(^11^C) [GBq] is the ^11^C radioactivity at end-of-bombardment (EOB), Y_SAT_(^11^C) [GBq/µA] is an empirical constant that is dependent on the nuclear reaction and proton energy, *t* [min] is the irradiation time, and *I* [µA] is the beam current. The half-life (T_½_) for ^11^C is 20.38 min (Sowby 1983). With 16.6-MeV protons, the value of Y_SAT_(^11^C) is 7.89 GBq/uA (IAEA database 2021), not corrected for partial pressure of hydrogen.

#### Target chamber and gas

**Fig. 1:**
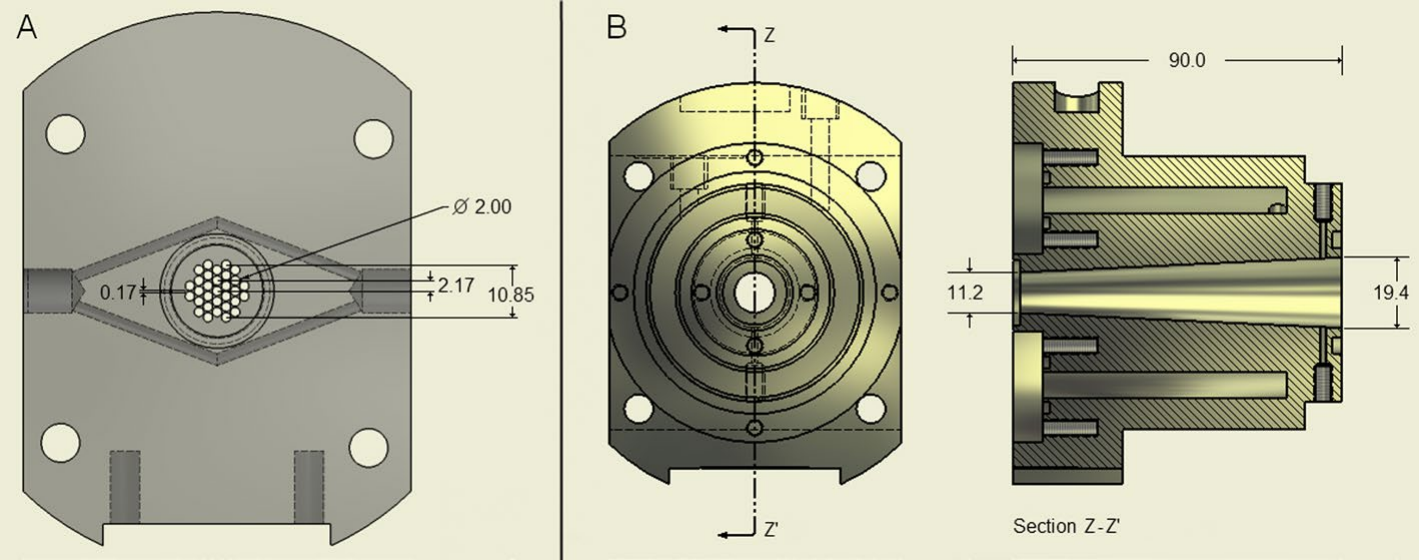
Target chamber. Dimensions in millimeters. **A** Front flange for target chamber. The grid structure and cooling channels drilled into the front piece are displayed in the cutaway drawing. **B** Front view (left) and cutaway drawing (right)

The aluminum target chamber had a conical shape, a cavity length of 90 mm, a front diameter of 11.2 mm, and a back diameter of 19.4 mm, for a target volume of 16.9 cm^3^ (Fig. 1). The inlet foil was 25-µm–thick stainless steel (AISI 321, Goodfellow Metals, England). The inlet aperture of the front piece was constructed as a gridded structure with 2-mm holes spaced by 0.17-mm walls, drilled into a separately cooled aluminum plate. The calculated transparency of this grid was 76%. The proton beam was shaped to a 10-mm diameter using a water-cooled collimator located 10 cm in front of the target chamber. The end of the target chamber was a separately cooled aluminum plate. The seals at the inlet foil and end of target were aluminized stainless steel C-rings (Garlock Helicoflex, Palmyra, NY, USA).

A jacket for cooling media was machined into the aluminum piece around the chamber. The cooling of this piece and the other separately cooled pieces was connected in a series. During the experiments, water was circulated at a flow of 3 L/min through the system, which included a heat exchanger (CFT-75, Thermo Fisher Scientific, Waltham, MA, USA) where the circulating water temperature could be set at 10–70 °C. The target chamber (Fig. 1B) temperature *T* [°C] was measured with a thermocouple drilled 6 mm deep into the outer surface of the chamber at the midway point of its length. The target chamber was connected to a thin film strain gauge pressure transducer (Trafag 8251, 0–100 bar, Trafag A.G., Zurich, Swizerland) and two pneumatically operated two-way valves (Swagelok, SS-41S1, Swagelok Company, Solon, Ohio, USA). These valves functioned as the target gas-filling valve and as the outlet valve for the gas. A line was then directed to the hot cell receiving the produced radioactivity. All filling and emptying lines were 1/16″ (1.58 mm) outer diameter and 1 mm inner diameter stainless steel lines (AISI 316).

A gas mixture of 99.8% N_2_–0.2% O_2_ (purity 99.999%, Linde Gases, Finland) was used for [^11^C]CO_2_ production, and a mixture of 95% N_2_–5% H_2_ (purity 99.999%, Woikoski OY, Finland) was used for [^11^C]CH_4_ production.

### Procedures

#### Irradiation categories

Irradiations were carried out in three categories for both [^11^C]CO_2_ and [^11^C]CH_4_ setups.

To begin with, a series of runs was done to investigate the repeatability and robustness of the experimental setup and procedures. In this first category, all irradiation parameters (*I* = 20 µA, *T* = 20 °C, *t* = 10 min) were kept unchanged (n = 10 for [^11^C]CO_2_ and n = 10 for [^11^C]CH_4_ irradiations).

The second category irradiations were for investigating [^11^C]CO_2_ and [^11^C]CH_4_ yields with a constant irradiation time of 20 min. The target chamber temperature was set at either 10, 40, or 70 °C, and the nominal beam current was set at 10, 20, 30, or 40 µA. Each measurement was performed twice in this category (n = 24 for [^11^C]CO_2_ and n = 24 for [^11^C]CH_4_ irradiations).

The third category was the charge–dose series performed only for [^11^C]CH_4_. The target chamber temperature was held constant at 40 °C. At three separate beam current settings of 10, 20, and 40 µA, the irradiation time was varied to deliver approximate irradiation doses of 120, 240, 360, 480, 600, and 800 µA∙min (n = 18 for [^11^C]CH_4_ irradiations).

Experiments in all categories were carried out in random order within the categories during a time span of about 1 year (n = 34 for [^11^C]CO_2_ and n = 52 for [^11^C]CH_4_ irradiations). Typically, two runs were done on each study day.

#### Target gas filling and emptying

Initially, the temperature of the heat exchanger that controlled the target chamber temperature was set to 20 °C. The target chamber was then filled with the gas mixture to a pressure of 35 bars and emptied. This cycle was repeated once and the target chamber was subsequently filled and closed off. After this step, the desired temperature (range 10–70 °C) was set on the heat exchanger, and the target chamber was allowed to reach this temperature. Irradiations were not started before a stable set temperature was reached. In this manner, the amount of the target molecules was the same for all irradiations, independent of pressure and temperature.

The irradiated target gas was released to a hot cell via a capillary and valve arrangement (Swagelok SS-41S1 and SS-41S2, Swagelok Company, Solon, Ohio, USA) at 200 mL/min. This flow was regulated by a mass flow controller (Bronkhorst F-201C-FA, Bronkhorst High-Tech B.V., Ruulo, Netherlands). After the initial emptying, the chamber was flushed by refilling it with the target gas mixture and releasing the gas to the same receiving hot cell. These two combined gas batches were the total collected and measured gas.

#### Collection and measurement of ^11^C radioactivity

The irradiated gas was passed through a trap, which was placed inside an isotope calibrator (Capintec CRC-15R, Capintec Inc., Ramsey, NJ, USA). Ascarite (20–30 mesh, Thomas Scientific, Swedesboro, NJ, USA) at ambient temperature was used to trap the produced [^11^C]CO_2_, and the [^11^C]CH_4_ was trapped in Porapak N (80–100 mesh, Waters Corp., Milford, MA, USA) at liquid argon temperature. After decay of the co-trapped ^10^C-species (T_½_ = 19.3 s) (Kondev et al. 2021), the reading from the isotope calibrator was taken as the amount of [^11^C]CO_2_ or [^11^C]CH_4_ produced. Gas exiting the trap was collected, and volume was measured. After the target emptying procedure, a representative 50-mL sample of the non-trapped gas was analyzed for absolute radionuclide content of ^11^C and ^13^N. Nitrogen-13 (T_½_ = 9.97 min) is formed through the nuclear reactions ^14^N(p,d)^13^N (threshold energy, E_thr_ = 11.44 MeV) (Beebe-Wang 2003) and ^16^O(p,α)^13^N (E_thr_ = 6.0 MeV) (IAEA-TECDOC-1211,2001).

### Calculations and notations

Determined saturation yields were calculated from measured radioactivities using Eq. 1.

Rearrangement of Eq. 1 gives

2 $$Y_{{{\text{SAT}}}} ({}_{{\text{~}}}^{{11}} C) = \frac{{A_{{{\text{EOB}}}} \left( {{}_{{\text{~}}}^{{11}} C} \right)}}{{\left( {1 - e^{{ - ln2 \cdot t/T_{{1/2}} }} } \right) \cdot I}}$$

We can then, in a similar manner, express

$$Y_{{{\text{SAT}}}} ({}_{{\text{~}}}^{{11}} CO{\text{2}}) = \frac{{A_{{{\text{EOB}}}} \left( {{}_{{\text{~}}}^{{11}} CO{\text{2}}} \right)}}{{\left( {1 - e^{{ - ln2 \cdot t/T_{{1/2}} }} } \right) \cdot I}}\;and\;Y_{{{\text{SAT}}}} ({}_{{\text{~}}}^{{11}} CH{\text{4}}) = \frac{{A_{{{\text{EOB}}}} \left( {{}_{{\text{~}}}^{{11}} CH{\text{4}}} \right)}}{{\left( {1 - e^{{ - ln2 \cdot t/T_{{1/2}} }} } \right) \cdot I}}$$

Here, A_EOB_(^11^CO_2_) stands for the measured radioactivity of [^11^C]CO_2_, and A_EOB_(^11^CH_4_) for the measured radioactivity of [^11^C]CH_4_, both at the EOB and in units of GBq. In a corresponding manner, Y_SAT_(^11^CO_2_) stands for the measured saturation yield of [^11^C]CO_2_, and Y_SAT_(^11^CH_4_) for the measured saturation yield of [^11^C]CH_4_ in units of GBq/µA.

#### Semi-theoretical target model

From a purely theoretical point of view, the saturation yields are expected to be constant over the range of irradiation time and particle beam intensity used in this study. However, although the Y_SAT_(^11^CO_2_) remained essentially constant, the value of Y_SAT_(^11^CH_4_) had a negative linear correlation with the beam current and positive correlation with the target chamber temperature. We propose a simple mathematical model for this behavior, expressed as:

2a $$Y_{{{\text{SAT}}}} ({}_{ }^{11} {\text{CH}}_{4} ) = a \cdot I + k$$

where *k* is the y-axis intercept of the linear fitted function at a certain gas temperature and *a* is the mean of the slopes of the linear fits at various temperatures. With this expression, we can analyze the *k* values as a function of the absolute temperatures at which they were measured and fit a linear function to the data points intercepting zero. This function is written as:

3 $$k = b \cdot T$$

where *b* is the slope of the fitted linear function. Combining Eqs. 2a and 3, we can then express:

2b $$Y_{{{\text{SAT}}}} ({}_{ }^{11} {\text{CH}}_{4} ) = a \cdot I + b \cdot T$$

Subsequently, we can express:

2c $$a \cdot I + b \cdot T = \frac{{cA_{{{\text{EOB}}}} \left( {{}_{{\text{~}}}^{{11}} {\text{CH}}_{4} } \right)}}{{\left( {1 - e^{{ - ln2 \cdot t/T_{{1/2}} }} } \right) \cdot I}}$$

and after rearrangement:

2d $$cA_{{{\text{EOB}}}} \left( {{}_{{\text{~}}}^{{11}} {\text{CH}}_{4} } \right) = \left( {a \cdot I^{2} + b \cdot T \cdot I} \right) \cdot \left( {1 - e^{{ - ln2 \cdot t/T_{{1/2}} }} } \right)$$

where *cA*_EOB_(^11^CH_4_) is now the model-predicted activity for [^11^C]CH_4_.

The constants *a* and *b* in Eq. 2b–d are not expected to be universal but rather are expected to depend on particular target chamber construction with regard to chamber materials, geometry, and temperature control.

### Statistical methods

Data are given as mean ± standard deviation (SD), and relative standard deviations (RSDs) were calculated. Target pressures during irradiations at various nominal beam currents (Additional file 1: Table S1) were examined using two-way analysis of variance (ANOVA) to estimate the effect of temperature and nominal beam current and their interaction on the target pressures. In addition to main effects, the simple main effects were examined further using Tukey’s post hoc test if significant main effects were identified in the data.

For the first category data of repeatability (Additional file 1: Table S2), a one-sample t-test was performed to compare the means of the measured radioactivities of A_EOB_(^11^CO_2_) and A_EOB_(^11^CH_4_) against their corresponding theoretical mean ^11^C yield A_EOB_(^11^C), which was calculated and decay-corrected for EOB based on the recorded irradiation values and normal distribution of the data. Furthermore, differences between the measured A_EOB_(^11^CO_2_) and A_EOB_(^11^CH_4_) in the first category were examined using unpaired t-tests without Welch’s correction, based on the similar variances of the data sets.

For the second category data (Additional file 1: Table S3), simple regression analysis was performed to examine the relationship between the measured and calculated values. For the A_EOB_(^11^CH_4_), this comparison was impossible because the data did not have a normal distribution, even after transformation. Differences in the measured A_EOB_(^11^CO_2_) and A_EOB_(^11^CH_4_) radioactivities were further examined using two-way ANOVA to estimate the effect of target chamber temperature and nominal current and their interaction on the measured radioactivities. In addition to main effects, the simple main effects were examined further using Tukey’s post hoc test if there were significant main effects in the data.

For the third category data (Additional file 1: Table S4), the definite integrals of the theoretical A_EOB_(^11^C) and the measured and predicted radioactivities for A_EOB_(^11^CH_4_) as a function of irradiation time were estimated with a simple area under the curve (AUC) method in order to investigate the specificity and selectivity of the model. Correlations between the measured and predicted radioactivities for A_EOB_(^11^CH_4_) were further examined with the Pearson correlation coefficient (r) to confirm model specificity.

Differences were considered significant if the *P* value was less than 0.05. All statistical analyses were performed using GraphPad Prism (version 8; GraphPad Software).

## Results

All irradiation parameters, measured radioactivities, and corresponding theoretical ^11^C yields, calculated using Eq. 1, are summarized in Additional file 1: Tables S1–S5. After standard loading, target gas pressure followed the temperature change as the intended setting was reached.

### System behavior

#### Target gas filling and irradiation pressures

Additional file 1: Table S1 presents the pressures before and during the irradiations as a function of temperature and beam current.

Both the target chamber temperature and the nominal current each had significant (*P* < 0.0001) single effects on the target pressure, with nominal current making the greater contribution when assessed from the F ratio. More detailed statistics, including differences across the simple main effects results from the Tukey’s post hoc test, are given in the Additional file 1.

For [^11^C]CO_2_ production, at 10 °C, the mean initial pressure was 34.6 ± 0.17 bar. Corresponding values for 40 °C and 70 °C were 38.2 ± 0.43 bar and 41.1 ± 0.53 bar, respectively, and results of single effect ANOVA were significant (*T* [F (2, 12) = 186.3, *P* < 0.0001], *I* [F (3, 12) = 1259, *P* < 0.0001]. No interaction effect was observed (*P* = 0.8903).

For [^11^C]CH_4_ production, at 10 °C, the mean initial pressure was 35.0 ± 0.15 bar. Corresponding values for 40 °C and 70 °C were 38.5 ± 0.12 bar and 41.7 ± 0.10 bar, respectively, and single effect ANOVA indicated significance (*T* [F (2, 12) = 42.84, *P* < 0.0001], *I* [F (3, 12) = 216.9, *P* < 0.0001]. No interaction effect was observed (*P* = 0.9201).

Overall, the highest target pressure during irradiation in [^11^C]CO_2_ and [^11^C]CH_4_ production was obtained using the greatest nominal current and target chamber temperature. Based on the consistent pressure rise, a thick target condition was maintained throughout the beam current and temperature range applied.

#### First category results and repeatability

The RSDs for run-time parameters (*I*,* T*,* t*) were 0.1–2.2%. The theoretical A_EOB_(^11^C) was calculated from the beam current and time, so that the A_EOB_(^11^C) RSD reflects the variation in the *I* and* t*, and RSD for the measured A_EOB_(^11^CO_2_) or A_EOB_(^11^CH_4_) indicates the variation in the whole system of targetry, delivery, and measurement. The RSD was 3.6% for the measured A_EOB_(^11^CO_2_) and 0.8% for the corresponding theoretical A_EOB_(^11^C). The RSD for measured A_EOB_(^11^CH_4_) was 2.8%, compared with 1.2% for the corresponding theoretical ^11^C A_EOB_(^11^C).

The measured mean A_EOB_(^11^CO_2_) of 24.0 ± 0.87 was significantly lower than the theoretical A_EOB_(^11^C) (*P* < 0.0001), as was also the case with the measured mean A_EOB_(^11^CH_4_) of 18.3 ± 0.52, compared with the corresponding theoretical A_EOB_(^11^C) (*P* < 0.0001). The A_EOB_(^11^CH_4_) was significantly lower than A_EOB_(^11^CO_2_) (*P* < 0.0001). The mean ratio of measured to theoretical was 0.70 ± 0.03 for [^11^C]CO_2_ and 0.54 ± 0.01 for [^11^C]CH_4_.

Overall, as the RSDs were clearly less than 5%, the system showed excellent repeatability. In addition, even in these moderate irradiation conditions, the measured A_EOB_(^11^CO_2_) and A_EOB_(^11^CH_4_) groups differed, and both measured groups differed from the theoretical A_EOB_(^11^C).

#### Radioactivity content in the non-trapped gas

In the non-trapped gas, the measured ^13^N content was a function of the beam current and Y_SAT_(^13^N), in analogy with Eq. 1. This behavior was similar between production of [^11^C]CO_2_ and [^11^C]CH_4_. The determined Y_SAT_(^13^N) was 0.867 ± 0.028 GBq/µA in the [^11^C]CO_2_ production (n = 24) and 0.783 ± 0.035 GBq/µA in the [^11^C]CH_4_ production (n = 24). These results reflect the dissimilarities in the gas composition for the different production methods and nuclear reactions available (see Methods; Target chamber and gas and Collection and measurement of ^11^C radioactivity).

^11^C content in the non-trapped gas was assumed to be in the form of [^11^C]CO. As expected, the non-trapped ^11^C content was relatively high from the N_2_-O_2_ target mixture, as the [^11^C]CO amount relative to the trapped [^11^C]CO_2_ was 1.48 ± 0.59%. Non-trapped ^11^C in the [^11^C]CH_4_ production was below the detection limit.

### Second category results: [^11^C]CO_2_ and [^11^C]CH_4_ yields

Second category results are tabulated in Additional file 1: Table S3, and additional results of the statistical analysis are given in the supplementary information. The ratio of measured A_EOB_(^11^CO_2_) to theoretical A_EOB_(^11^C) was practically constant across the studied range of beam current and temperature, and the ratio also was the same as in the first category results. In addition, this steady dependency was seen as a strong correlation for A_EOB_(^11^CO_2_) as a function of A_EOB_(^11^C): the fitted regression model for A_EOB_(^11^CO_2_) was Y = 0.6301X + 3.528, and the overall regression was significant (R^2^ = 0.99, *P* < 0.0001).

In contrast, as noted in the statistical methods, regression analysis could not be conducted with the A_EOB_(^11^CH_4_) and A_EOB_(^11^C) data. Furthermore, the ratio of measured A_EOB_(^11^CH_4_) to theoretical A_EOB_(^11^C) was not constant and had a negative correlation with increasing beam current and positive correlation with increasing target chamber temperature within all nominal current groups.

**Fig. 2:**
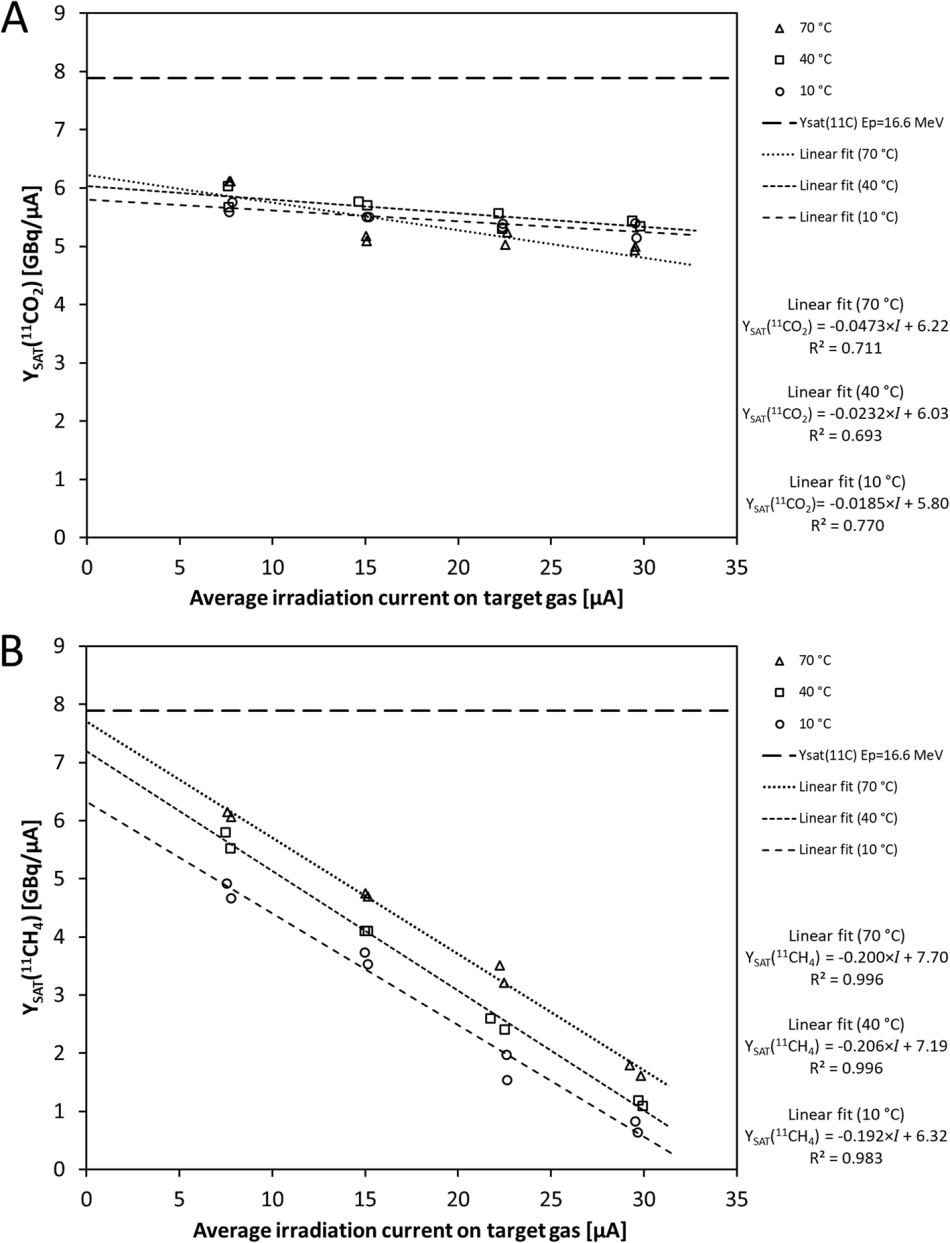
Saturation yields, Y_SAT_ [GBq/µA], calculated from the measured second category data (Additional file 1: Table S3) as a function of target chamber temperature and beam current. Dashed lines show linear fit for Y_SAT_(^11^CO_2_) and Y_SAT_(^11^CH_4_) at various target chamber temperatures. The theoretical saturation yield of ^11^C for the given proton energy, uncorrected for the N_2_-H_2_ composition, is indicated with a horizontal dashed line. **A** Saturation yield of ^11^C-carbon dioxide, i.e., Y_SAT_(^11^CO_2_). **B** Saturation yield of ^11^C-methane, i.e., Y_SAT_(^11^CH_4_). The y-axis intersections of the linear fit equations (constant term *k*) were used to determine the constant *b* in Eq. 2b

The same pattern was seen when using saturation yields, Y_SAT_. The Y_SAT_(^11^CO_2_) and Y_SAT_(^11^CH_4_) were calculated from the second category data using Eq. 2 (Additional file 1: Table S3). Figure 2 depicts the Y_SAT_(^11^CO_2_) and Y_SAT_(^11^CH_4_) plotted against beam current at varied target chamber temperatures. Y_SAT_(^11^CO_2_) was essentially unchanged by the beam current and target chamber temperature, as shown in Fig. 2A and tabulated in Additional file 1: Table S5, with a small SD (0.30) for the whole sample (pooled mean, 5.47 GBq/µA). In contrast, Y_SAT_(^11^CH_4_) showed a linear proportionality to the beam current with a negative slope, and the trendlines of the Y_SAT_(^11^CH_4_) shifted vertically (Fig. 2B) with target chamber temperature.

There was a significant interaction between the effects of the beam current and target chamber temperature on A_EOB_(^11^CO_2_) (*P* = 0.0145), and they each singly had a significant effect on the A_EOB_(^11^CO_2_) (temperature, *P* = 0.0008; beam current, *P* < 0.0001). Additional results of the Tukey’s post hoc test are presented in the supplementary information. Briefly, no trend was observed across the sample despite a significant effect of target chamber temperature in two sets in the multiple comparison. The beam current was the major factor in the A_EOB_(^11^CO_2_).

For the A_EOB_(^11^CH_4_), temperature and beam current also interacted significantly in affecting measured radioactivities (*P* = 0.0013), while each alone also affected A_EOB_(^11^CH_4_) significantly (both *P* < 0.0001). Additional results of the Tukey’s post hoc test are presented in the Additional file 1. Briefly, higher radioactivities within a group of beam currents were seen at higher temperatures. Interestingly, the highest radioactivity was measured at 30 µA, whereas the measured A_EOB_(^11^CH_4_) at 40 µA could not exceed any A_EOB_(^11^CH_4_) at the corresponding target chamber temperature setting in other beam current groups. Furthermore, a curvilinear relationship was found for A_EOB_(^11^CH_4_) and beam current, where the curvature maximum was shifted towards higher beam current with increasing target chamber temperature.

### Semi-theoretical target model

In an ideal situation, [^11^C]CH_4_ is formed quantitatively from produced ^11^C, resulting in identical Y_SAT_(^11^C) and Y_SAT_(^11^CH_4_). These saturation yields would also be independent from the parameters of beam current and irradiation time (Eq. 1). However, the systematic observations indicate that the opposite is the case.

**Fig. 3:**
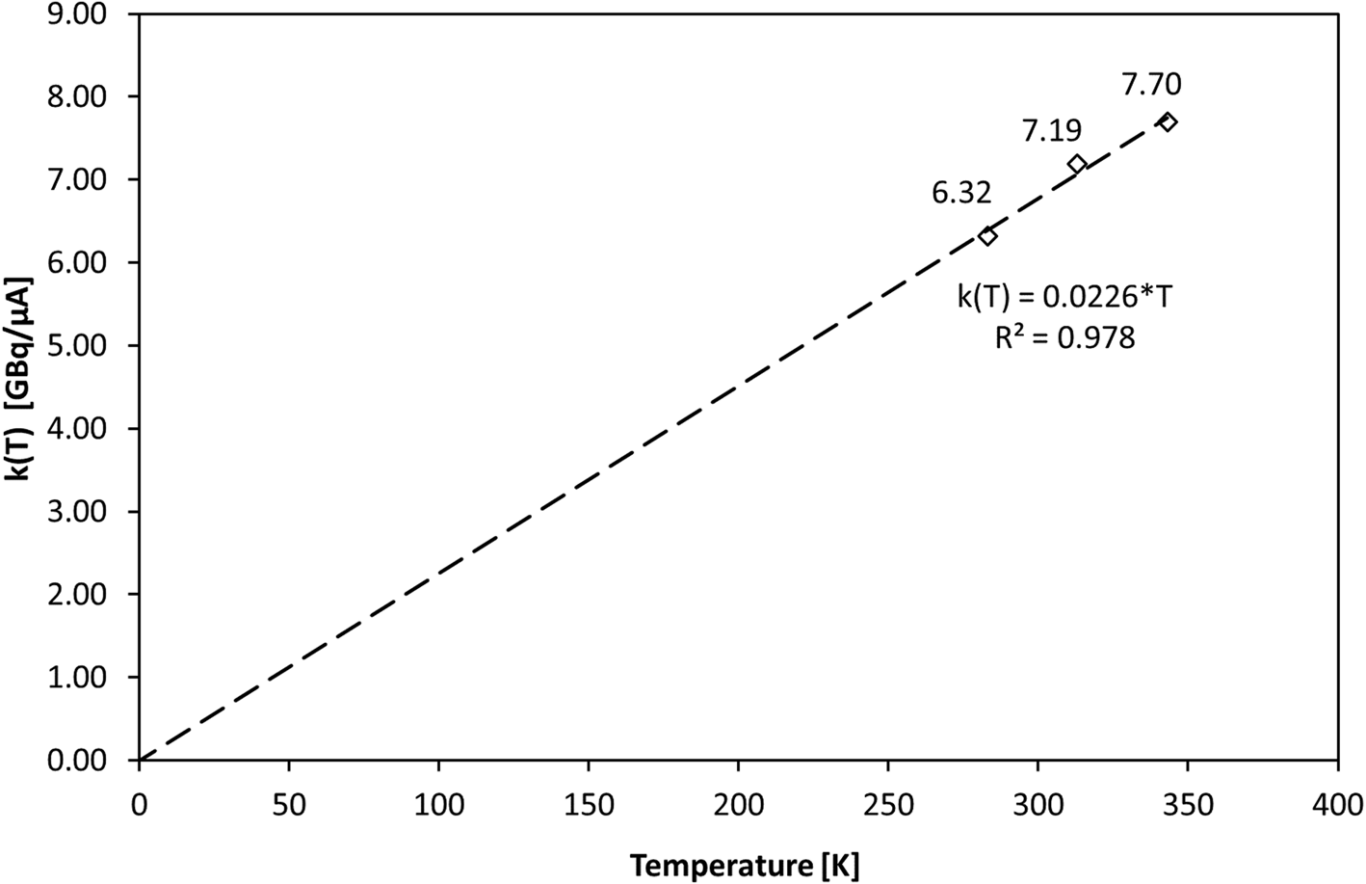
Temperature-dependent factor k(T) for [^11^C]CH_4_ production. Scatter plot data points are from the experimental Y_SAT_(^11^CH_4_) data (Fig. 2B), incorporating the constant terms *k* of the linear-fit equations. The linear fit here is forced through zero, and the slope gives the constant *b* (Eq. 2b)

The y-axis intersection point in Fig. 2B is the constant term *k* in linear Eq. 2a. Figure 3 shows the constants *k* from Fig. 2B plotted against the absolute temperature, producing a good fit through zero (R^2^ = 0.978). From these data, we can derive values for constants *a* and *b* using a linear fit:

$$\begin{aligned}&a = \, - 0.{\text{199 GBq}}/\upmu {\text{A}}^{{2}} \\ &b = \, 0.0{\text{226 GBq}}/\left( {\upmu {\text{A}}\cdot{\text{K}}} \right)\end{aligned}$$

where *a* is the constant in Eq. 2a and the mean of the slopes in Fig. 2B, and b is the constant in Eq. 3 and the slope in Fig. 3.

#### Third category results: charge dose series with model-predicted activities

**Fig. 4:**
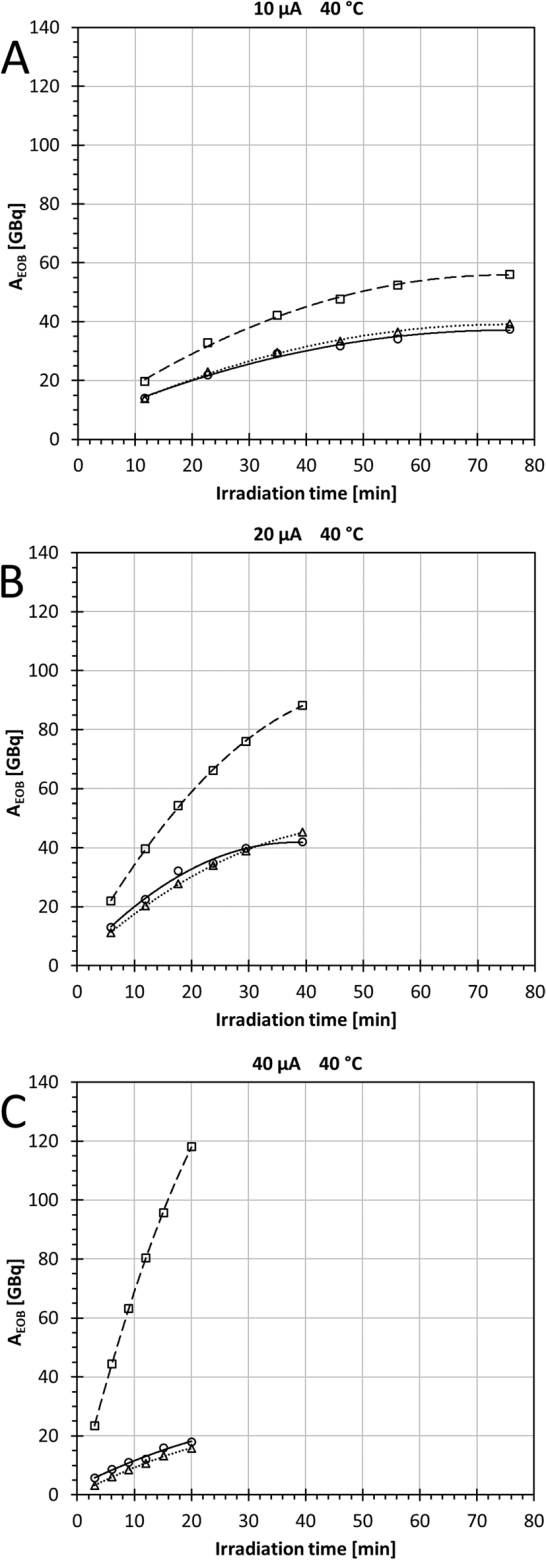
Radioactivity at EOB as a function of irradiation time at a constant 40 °C target chamber temperature for nominal beam currents: **A** 10 µA, **B** 20 µA, and **C** 40 µA. Theoretical ^11^C radioactivity A_EOB_(^11^C) (□), measured [^11^C]CH_4_ radioactivity A_EOB_(^11^CH_4_) (○), and predicted ^11^CH_4_ radioactivity cA_EOB_(^11^CH_4_), calculated according to model Eq. 2d (∆)

Figure 4 presents the third category experiments, where the target chamber temperature was held constant and beam current had three settings: 10, 20, or 40 µA. Theoretical A_EOB_(^11^C), model-predicted cA_EOB_(^11^CH_4_), and measured A_EOB_(^11^CH_4_) yields were plotted against irradiation time.

As can be seen, the semi-theoretical model (Eq. 2d) shows an excellent fit to the third category results (Fig. 4, Additional file 1: Table S4). The definite integrals (AUCs) of the measured A_EOB_(^11^CH_4_) and predicted cA_EOB_(^11^CH_4_) showed that the model could estimate the measured A_EOB_(^11^CH_4_) with > 95% accuracy at 10 and 20 µA, and with a 77% correspondence at 40 µA. The measured and predicted radioactivities for A_EOB_(^11^CH_4_) were strongly correlated at 10 µA (r(4) = 0.9979; *P* < 0.0001). A similarly strong correlation was observed at 20 µA (r(4) = 0.9846; *P* = 0.0004) and at 40 µA (r(4) = 0.9846; *P* < 0.0001).

An increasing divergence of the measured [^11^C]CH_4_ from the theoretical ^11^C yield as a function of raised beam current was evident. The AUCs of the A_EOB_(^11^CH_4_) against A_EOB_(^11^C) were 33% lower at 10 µA, 45% lower at 20 µA, and 83% lower at 40 µA.

## Discussion

### System behavior

Pressure behavior followed the general gas law when bringing the gas from loading to the specific initial temperature. During the irradiation, the pressures were much higher than the ideal gas law suggests from the heat exchanger temperatures (target chamber). This is in line with previously published findings of heterogeneous density and temperature conditions (Jahangiri et al. 2016; Heselius et al. 1982a; Köble et al. 1989).

### Second category: [^11^C]CH_4_ yields

Even though the Y_SAT_(^11^CO_2_) values did not reach the theoretical level, this serves as a reference level for the [^11^C]CH_4_ production process. According to Buckley et al. (2000), [^11^C]CO_2_ yields from similar high pressure targets are 84–90% of theoretical values. Regarding the [^11^C]CH_4_ yields, particularly at higher beam currents, a considerable amount of the produced ^11^C-radioactivity was not received into the hot cell. Similar behavior has been reported broadly by other groups (Andersson et al. 2009; Buckley et al. 2000; Koziorowski et al. 2010). Flushing the target chamber and transfer line with target gas or repeated irradiation of fresh target gas did not increase the received radioactivity, but more [^11^C]CH_4_ was received as a function of elevated target chamber temperature.

### Semi-theoretical target model

Comparing the *t* = 20 min data points at the three beam current settings (Fig. 4A–C), the behavior of the yield values were similar to the behavior reported by Andersson et al. (2009), who described their approach to empirically pinpointing an optimal beam current and irradiation time. In the current work, a clear increase in yield (either cA_EOB_(^11^CH_4_) or A_EOB_(^11^CH_4_)) was observed from 10 to 20 µA, but the 40 µA yield was even lower than yield at 10 µA.

**Fig. 5:**
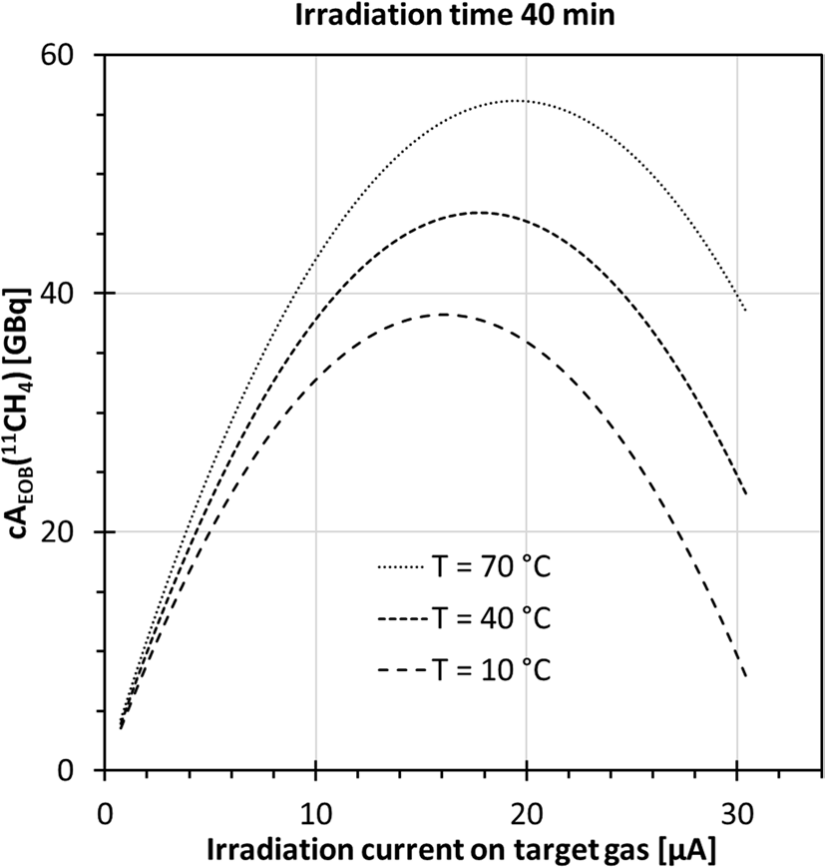
Predicted ^11^CH_4_ radioactivity cA_EOB_(^11^CH_4_) for 40-min irradiation across a beam current range at various target chamber temperatures, calculated according to model Eq. 2d

The graph of the model equation for varying beam current is a downward opening parabola. Figure 5 shows calculated graphs for 40-min irradiation for *T* = 10, 40, or 70 °C (Eq. 2d). The apex of the parabola indicates the optimal beam current for a chosen irradiation time. Elevation in the target chamber temperature shifted the whole curve to greater yields and moved the apex towards higher beam currents. This pattern agrees well with the curvilinear relationship found for beam current and measured A_EOB_(^11^CH_4_) with the *T* shift (see second category results for [^11^C]CO_2_ and [^11^C]CH_4_ yields).

### Considerations of the divergent [^11^C]CH_4_ production

Overall, the obtained radioactivity yield data from the identical irradiation conditions of the first and second categories show a clear difference between the N_2_–O_2_ target performance and that of N_2_–H_2_, where the only distinguishing factor was the additive gas in the bulk target nitrogen. Even though the exact nature of the radiolytic or chemical reactions occurring during the irradiation of the nitrogen–oxygen mixture is not entirely known (Christman et al. 1975), it is established that the reactivity of ^11^C atoms with oxygen is about one order of magnitude higher than with nitrogen (Ache and Wolf 1966). [^11^C]CO_2_ is obtained as a secondary product from primary [^11^C]CO by radiolytic oxidation (Wolf and Redvanly 1977; Elias and Wolf 1968), whereas for [^11^C]CH_4_, the production proceeds via a [^11^C]CN* intermediate before radiolytic reduction with hydrogen (Ache and Wolf 1966; Christman et al. 1975).

The findings strongly imply that the experimental system is not responsible for deviation from the theoretical yields beyond what is observed with [^11^C]CO_2_ production. Obviously, the sensitivity of secondary hot-atom processes in these two target systems is crucially different in terms of the effects of irradiation intensity. It is worth noting that the Y_SAT_(^11^CH_4_) at nominal 10 µA irradiation could be restored to the Y_SAT_(^11^CO_2_) level by increasing the target chamber temperature (Fig. 2). Thus, the waning behavior of Y_SAT_(^11^CH_4_) can be interpreted to arise from an in-target phenomenon that limits the reaction of the ^11^CN* intermediate with hydrogen, involving a mechanism with a thermodynamic relation to the target chamber surface.

#### Ammonia formation and the wall effect

In the energetic conditions, various side reactions between the target gas components (nitrogen and hydrogen) can be anticipated. The work that examined the ammonia formation (Buckley et al. 2000) measured NH_3_ from the irradiated gas. Detected quantities quickly reached a non-changing state, which was inferred to reflect equilibrium conditions of ammonia, hydrogen, and nitrogen. These measurements were done with a 20 µA proton beam only, with results showing that the [NH_3_]/[H_2_] concentration ratio reached a constant value of 0.05 for a setup closest to our design, i.e., a conical aluminum target chamber. Thus, the observations of low and declining methane yields are not explained by this observation.

**Fig. 6:**
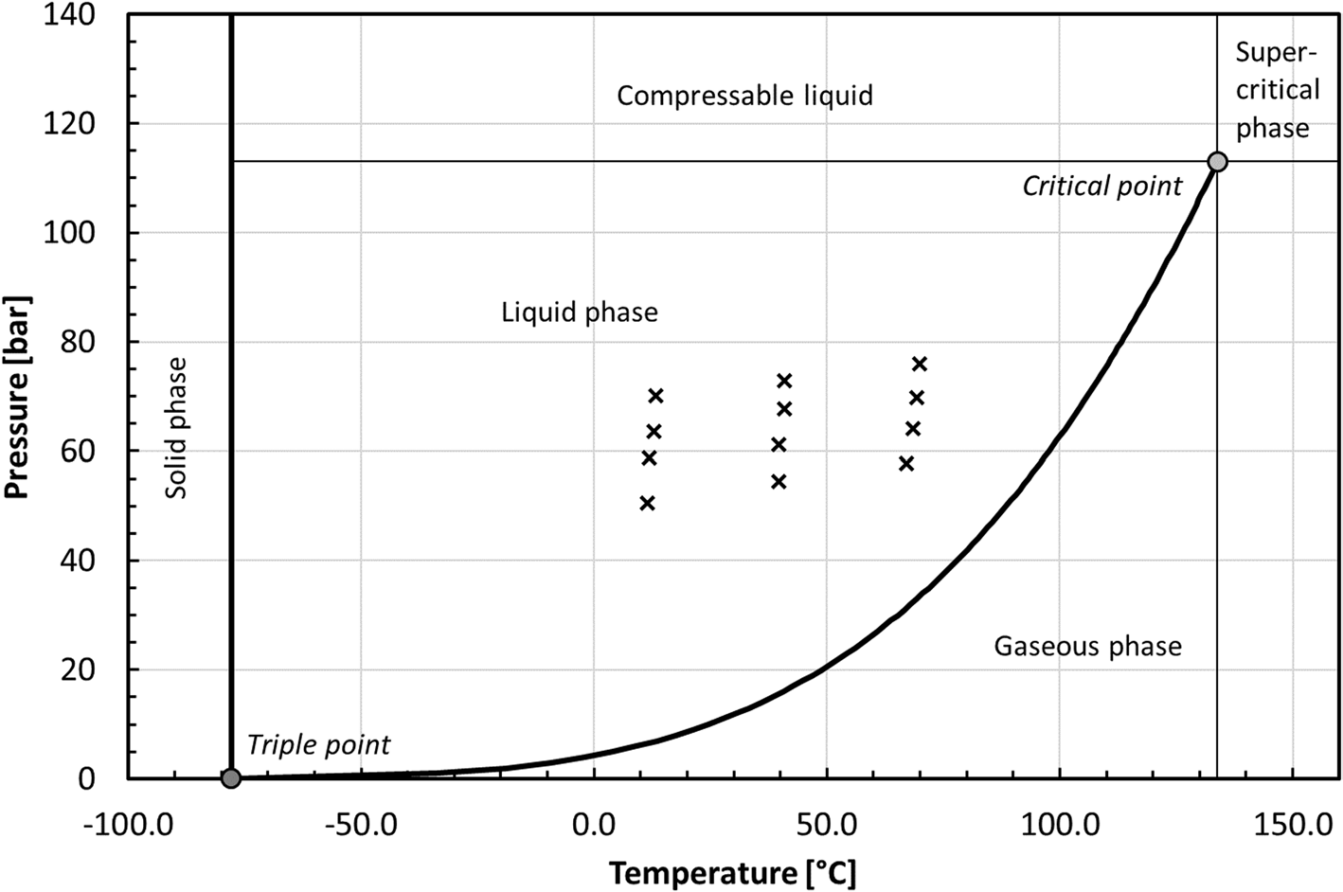
Ammonia phase diagram. Irradiation conditions (×) of [^11^C]CH_4_ production in this study, i.e., beam-on pressure and target chamber temperature

Whether the produced compound would be in gaseous phase depends on the pressure and temperature conditions in the target chamber. The critical point of CH_4_ is 46 bar and − 82.6 °C (Dean 1999), implying a gaseous or supercritical phase at higher temperatures regardless of the target pressure. However, in our target conditions, the generated NH_3_ is in liquid phase (Lange 1968) (Fig. 6), suggesting that most of the ammonia resided condensed on the target walls. Effectively, the hydrogen bound to ammonia in liquid state would not be available for the secondary hydrogenation reaction of CN* to CH_4_.

Dissociation of the bonds of both reactant molecules is needed in the Haber–Bosch process, where the triple bond of the nitrogen molecule is the rate-limiting step requiring the high temperatures, which sets the activation energy. Electrochemical ammonia synthesis and plasma activation of the N_2_ molecule have gained attention as alternative nitrogen fixation methods (Rouwenhorst et al. 2020). Plasma conditions also are found in the target gas because ionization, dissociation, and excitation arise from the proton beam interaction (Hällsten et al. 2004). In addition, the target gas is affected by the energetic electrons that are produced, which have been investigated in terms of energy distribution and yield of ejected primary and secondary electrons (Hällsten and Solin 2004).

#### Temperatures of the target gas and the target chamber

From the measured pressures, the corresponding target gas temperatures during irradiation can be calculated using the relation of the general gas law pV = nRT.

The resulting temperatures are approximately 145–340 °C at the nominal current range of 10–40 µA and a 10 °C heat exhanger setting, 175–380 °C at the 40 °C setting, and 210–405 °C at the 70 °C setting.

These values are much higher than the measured target chamber temperatures, which implies effective heat conduction from the target gas to the aluminum target chamber and further to the stream of the cooling medium. Yet these temperatures are clearly in the gaseous region of the ammonia phase diagram for all measured pressures (Fig. 6), raising the question of whether ammonia condensation on the chamber surface can take place.

Earlier optical studies on the target gas behavior in a particle beam, however, give evidence of localized heat dissipation to the beam interaction volume and strong upward convection of the heated gas (Heselius et al. 1984, 1982b; Hällsten and Solin 2002; Solin et al. 1984). In an optical emission study, Heselius et al. (1984) reported light emission measurements directly from the beam interaction volume within the target gas. An emission line profile as a function of target gas density change was found, and based on that profile, a further relation, d*T/*d*I* = 15.4 K/µA, was derived, which allowed for temperature estimations. With that dependency, the temperature estimations in the current study would be approximately 130–470 °C at the 10 °C setting, 155–500 °C at the 40 °C setting, and 190–530 °C at the 70 °C setting. Even though the numerical value of the d*T/*d*I* relation corresponds to conditions specific to that study, there is a surprisingly good agreement with the temperatures obtained with the general gas law, especially at low beam current values in all heat exhanger settings. The difference in the values at high beam currents, on the other hand, is another indication of effective conduction of heat to the circulating fluid. Dissipated heat from the proton beam reached 680 W, and the maximum cooling capacity of the heat exchanger was 1900 W at 20 °C.

Interferograms and light emission photographs obtained directly from the irradiated gas in a windowed target chamber (Heselius et al. 1982b; Solin et al. 1984) show fringe patterns indicating a heterogenous heat map, strong upward flow of heated gas, and accompanying asymmetric density reduction in the beam interaction volume. Optically measured evidence of an uneven distribution of density reduction and heat also has been confirmed by measurements of radioactivity attached to the surface of a removable liner placed inside a target chamber (Solin et al. 1984). The determined amounts of activity produced by the proton beam were remarkably localized to the upper parts.

Conclusively, the excellent heat conduction properties of an aluminum target chamber coupled with the surrounding cooling jacket create such an effective heat sink that a target chamber temperature can be maintained 340 °C or 460 °C lower than temperatures calculated using pV = nRT or d*T/*d*I* = 15.4 K/µA. Given also the proof of upward and uneven distribution of heated gas, we have good reason to assume, particularly at lower chamber regions, that such temperatures allow for ammonia condensation on the surface.

### Limitations of the study

The setup did not allow for quantitative measurement of the ammonia during or post irradiation.

The distribution and chemical form of the non-received ^11^C radioactivity remains unknown.

## Conclusions

[^11^C]CH_4_ production can be improved by increasing the target chamber temperature, which is presented here in a mathematical formula. The second order equation reflects a limiting mechanism for [^11^C]CH_4_ that outweighs the ^11^C gain from the plain nuclear reaction with increasing beam current. The effect presents a challenge to the pursuit of high [^11^C]CH_4_ yield and, consequently, molar radioactivity.

Elevation in the target chamber temperature shifts the whole [^11^C]CH_4_ production to higher yields. Additionally, the optimal beam current at a certain irradiation time shifts towards a higher beam current.

A wall effect related to liquid ammonia implies the need to maintain lower irradiation pressures and higher target chamber temperature to decrease hydrogen escaping from the gas phase. In practice, adding volume and length to the target chamber geometry would facilitate lower pressures and increase the hydrogen reserve.

In summary, for high-yield [^11^C]CH_4_ production, we have identified and modeled target parameters that reduce the adverse wall effect and promote use of higher beam currents. Consequently, higher starting activities would offer benefit for a subsequent radiosynthesis in terms of improved product yield and molar radioactivity.

### Supplementary Information

**Additional file 1**: Contains supplementary materials, methods, and results.

## Data Availability

The datasets used and/or analyzed during the current study are available from the corresponding author on reasonable request.

## Abbreviations

^10^C: Carbon-10
^11^C: Carbon-11
^13^N: Nitrogen-13
^14^N: Nitrogen-14
A_EOB_: Radioactivity at EOB
ANOVA: Analysis of variance
AUC: Area under the curve
cA_EOB_: Predicted radioactivity at EOB
EOB: End-of-bombardment
E_thr_: Threshold energy
eV: Electron volt
*I*: Beam current
RSD: Relative standard deviation
SD: Standard deviation
*t*: Irradiation time
*T*: Target chamber temperature
Y_SAT_: Saturation yield; an empirical constant, dependent on the nuclear reaction and proton energy
