# Supplementary material for: In-target production of [11C]CH4 from a nitrogen/hydrogen gas target as a function of beam current, irradiation time, and target temperature
Source: EJNMMI Radiopharm Chem. 2024 Mar 25;9:24. doi: 10.1186/s41181-024-00255-1 (PMC11339016; doi:10.1186/s41181-024-00255-1)
Supplement: Supplementary file 1 — Additional file 1: Contains supplementary materials, methods, and results. [file 41181_2024_255_MOESM1_ESM.docx]

**Supplementary Information for**

In-target Production of [^11^C]CH_4_ from a Nitrogen/Hydrogen Gas Target as a Function of Beam Current, Irradiation Time, and Target Temperature

**Semi Helin^1^, Johan Rajander^2^, Jussi Aromaa^2^, Eveliina Arponen^1^, Jatta S. Helin^3,4^ and Olof Solin^1,2,5,6^**

^1^Turku PET Centre, Radiopharmaceutical chemistry laboratory, University of Turku, Kiinamyllynkatu 4-8, FI-20520 Turku, Finland

^2^Turku PET Centre, Accelerator laboratory, Åbo Akademi University, Kiinamyllynkatu 4-8, FI-20520 Turku, Finland

^3^MediCity Research Laboratory, University of Turku, Tykistökatu 6 A, FI-20520 Turku, Finland

^4^Turku PET Centre, Preclinical Imaging Laboratory, University of Turku, Tykistökatu 6 A, FI-20520 Turku, Finland

^5^Department of Chemistry, University of Turku, Henrikinkatu 2, FI-20500 Turku, Finland

^6^Turku PET Centre, Turku University Hospital, Kiinamyllynkatu 4-8, FI-20520 Turku, Finland

Contents

[**Table S1**. Target pressures during irradiations at various nominal beam currents. 2](#_Toc155656413)

[Detailed statistical results on the Table S1 data; effect of nominal current and temperature on target pressures during irradiations for [^11^C]CO_2_ and [^11^C]CH_4_ production 2](#_Toc155656414)

[**Table S2**. Measured data from the first category; repeatability 3](#_Toc155656415)

[**Table S3**. Measured data from the second category. 4](#_Toc155656416)

[Additional statistical results on Table S3 data; second category, [^11^C]CO_2_ and [^11^C]CH_4_ yields 5](#_Toc155656417)

[**Table S4**. Measured data from the third category. 6](#_Toc155656418)

[**Table S5**. Means and standard deviations (SD) of saturation activities Y_SAT_ [GBq/µA] at temperatures 10 °C, 40 °C and 70 °C. 7](#_Toc155656419)

# **Table S1**. Target pressures during irradiations at various nominal beam currents.

Mean values with standard deviations (N=8) before irradiations (zero current). Target was loaded to 35 bar pressure while the heat exchanger setting was 20 °C. After loading, the setting was changed to the desired value. Zero current values were recorded when the chamber body reached the temperature. Irradiation time was 20 min for all experiments. Data is from the second category irradiations.

| **Target pressure**  **[bar]** | | **[^11^C]CO_2_ production** | | | **[^11^C]CH_4_ production** | | |
| --- | --- | --- | --- | --- | --- | --- | --- |
| Nominal current  [μA] | | 10 °C | 40 °C | 70 °C | 10 °C | 40 °C | 70 °C |
| 0 | Mean | 34.6 | 38.2 | 41.1 | 35.0 | 38.5 | 41.7 |
|  | SD | 0.17 | 0.43 | 0.53 | 0.15 | 0.12 | 0.10 |
| 10 | | 51.16 | 53.74 | 56.75 | 50.76 | 54.99 | 57.99 |
|  |  | 50.20 | 53.86 | 57.49 | 50.12 | 53.91 | 57.33 |
| 20 | | 58.43 | 62.16 | 65.20 | 59.22 | 61.95 | 64.71 |
|  |  | 58.86 | 62.83 | 65.58 | 58.28 | 60.28 | 63.45 |
| 30 | | 64.74 | 68.89 | 72.11 | 64.51 | 68.79 | 68.94 |
|  |  | 66.52 | 70.09 | 71.95 | 62.66 | 66.40 | 70.37 |
| 40 | | 73.43 | 77.29 | 79.07 | 68.62 | 71.02 | 74.95 |
|  |  | 73.35 | 78.21 | 80.91 | 71.39 | 74.51 | 76.97 |

## Detailed statistical results on the Table S1 data; effect of nominal current and temperature on target pressures during irradiations for [^11^C]CO_2_ and [^11^C]CH_4_ production

**[^11^C]CO_2_**

ANOVA; there was a statistically significant single effect of temperature [F (2, 12) = 186.3, P < 0.0001] as well as nominal current [F (3, 12) = 1259, P < 0.0001] on the target pressure, but no interaction effect was observed (P = 0.8903).

Detailed differences across the simple main effect of temperature was examined after multiple comparison, which demonstrated significantly higher target pressures at 70 °C compared to 10 °C or 40 °C regardless the nominal current (ranging from P < 0.0001 to P = 0.0165).

Tukey’s *post hoc* test; the simple main effect of nominal current was examined, which showed that significantly higher target pressures were obtained at 40 µA compared to lower nominal currents regardless of the temperature (P < 0.0001) in the [^11^C]CO_2_ production.

**[^11^C]CH_4_**

ANOVA; there was a statistically significant single effect of temperature [F (2, 12) = 42.84, P < 0.0001] as well as nominal current [F (3, 12) = 216.9, P < 0.0001] on the target pressure, but no interaction effect was observed (P = 0.9201).

Detailed differences across the simple main effect of nominal current was examined after multiple comparison, which demonstrated significantly higher target pressures at 40 μA compared to lower nominal currents at all temperatures (ranging from P < 0.0001 to P = 0.0156).

Tukey’s *post hoc* test; the simple main effect of temperature on the target pressure was examined, which showed that at 10 μA nominal current, significantly lower target pressures were obtained at 10 °C compared to 40 °C (P = 0.0268) or 70 °C (P = 0.0004). When nominal current was adjusted to 20 μA, significantly higher target pressure was obtained at 70 °C compared to 10 °C (P = 0.0046). At nominal current of 30 μA, significantly lower target pressures were obtained at 10 °C compared to 40 °C (P = 0.0269) or 70 °C (P = 0.0017). Finally at 40 μA, significantly higher target pressure was obtained at 70 °C compared to 10 °C (P = 0.0020).

# **Table S2**. Measured data from the first category; repeatability

Collected radioactivities, A_EOB_(^11^CH_4_) and A_EOB_(^11^CO_2_), are decay corrected to the EOB. *I* is the irradiation current, measured from the target grid and body. To get the average irradiation current on the target gas, *I* is corrected by the grid factor. *T* is the measured target chamber temperature, and *t* is the recorded irradiation time. Theoretical A_EOB_(^11^C) is calculated based on the grid corrected irradiation values. Mean, Standard deviation (SD) and Relative standard deviation (RSD) are calculated for the repeated runs.

| **1^st^ cat.** | **[^11^C]CO_2_** | | | | | | **[^11^C]CH_4_** | | | | | |
| --- | --- | --- | --- | --- | --- | --- | --- | --- | --- | --- | --- | --- |
|  |  |  |  | Meas. | Theor. |  |  |  |  | Meas. | Theor. |  |
|  | ***I*** | ***T*** | ***t*** | ***A*_EOB_(^11^CO_2_)** | ***A_EOB_*(^11^C)** | ***Meas./theor.*** | ***I*** | ***T*** | ***t*** | ***A*_EOB_(^11^CH_4_)** | ***A_EOB_*(^11^C)** | ***Meas./theor.*** |
|  | [μA] | [°C] | [min] | [GBq] | [GBq] |  | [μA] | [°C] | [min] | [GBq] | [GBq] |  |
|  | 19.9 | 21.4 | 9.98 | 22.7 | 34.3 | 0.66 | 19.0 | 21.7 | 10.07 | 18.5 | 33.0 | 0.56 |
|  | 20.1 | 21.4 | 10.00 | 23.3 | 34.7 | 0.67 | 19.9 | 21.7 | 10.00 | 18.6 | 34.3 | 0.54 |
|  | 20.0 | 21.3 | 9.98 | 23.5 | 34.5 | 0.68 | 19.6 | 21.7 | 9.98 | 18.2 | 33.9 | 0.54 |
|  | 19.7 | 21.4 | 10.00 | 23.4 | 34.1 | 0.69 | 19.6 | 21.6 | 10.00 | 17.8 | 34.0 | 0.52 |
|  | 19.8 | 21.3 | 9.98 | 24.3 | 34.2 | 0.71 | 19.6 | 21.6 | 10.02 | 17.8 | 33.9 | 0.53 |
|  | 19.8 | 21.3 | 9.98 | 23.6 | 34.2 | 0.69 | 19.6 | 21.4 | 10.00 | 18.2 | 33.9 | 0.54 |
|  | 19.6 | 21.4 | 9.97 | 25.1 | 33.8 | 0.74 | 19.6 | 21.4 | 10.00 | 18.2 | 33.9 | 0.53 |
|  | 19.6 | 21.4 | 10.00 | 25.3 | 33.9 | 0.75 | 19.9 | 20.1 | 9.95 | 19.4 | 34.2 | 0.57 |
|  | 19.9 | 21.4 | 10.00 | 24.9 | 34.3 | 0.73 | 19.9 | 21.6 | 9.98 | 18.5 | 34.4 | 0.54 |
|  | 19.7 | 21.3 | 10.00 | 24.3 | 34.1 | 0.71 | 19.3 | 21.4 | 10.00 | 17.7 | 33.4 | 0.53 |
| **Mean** | **19.8** | **21.4** | **9.99** | **24.0** | **34.2** | **0.70** | **19.6** | **21.4** | **10.00** | **18.3** | **33.9** | **0.54** |
| **SD** | **0.17** | **0.05** | **0.01** | **0.87** | **0.26** | **0.03** | **0.28** | **0.48** | **0.03** | **0.52** | **0.42** | **0.01** |
| **RSD (%)** | **0.8** | **0.2** | **0.1** | **3.6** | **0.8** | **4.2** | **1.4** | **2.2** | **0.3** | **2.8** | **1.2** | **2.7** |

# **Table S3**. Measured data from the second category.

Collected radioactivities, A_EOB_(^11^CH_4_) and A_EOB_(^11^CO_2_), are decay corrected to the EOB. *I* is the irradiation current, measured from the target grid and body. To get the average irradiation current on the target gas, *I* is corrected by the grid factor, see Fig. 2. *T* is the measured target chamber temperature, and *t* is the recorded irradiation time. Theoretical A_EOB_(^11^C) is calculated based on the grid corrected irradiation values.

| **2^nd^ cat.** | | **[^11^C]CO_2_** | | | | | | **[^11^C]CH_4_** | | | | | |
| --- | --- | --- | --- | --- | --- | --- | --- | --- | --- | --- | --- | --- | --- |
| Nominal | |  |  |  | Meas. | Theor. |  |  |  |  | Meas. | Theor. |  |
| ***current*** | ***temperature*** | ***I*** | ***T*** | ***t*** | ***A*_EOB_(^11^CO_2_)** | ***A_EOB_*(^11^C)** | ***Meas./theor.*** | ***I*** | ***T*** | ***t*** | ***A*_EOB_(^11^CH_4_)** | ***A_EOB_*(^11^C)** | ***Meas./theor.*** |
| [μA] | [°C] | [μA] | [°C] | [min] | [GBq] | [GBq] |  | [μA] | [°C] | [min] | [GBq] | [GBq] |  |
| 10 | 10 | 10.3 | 11.2 | 20.00 | 22.2 | 30.5 | 0.73 | 10.2 | 11.7 | 20.02 | 17.9 | 30.3 | 0.59 |
|  |  | 10.1 | 11.3 | 20.00 | 21.1 | 29.8 | 0.71 | 9.9 | 11.3 | 20.00 | 18.3 | 29.4 | 0.62 |
|  | 40 | 10.1 | 39.2 | 19.98 | 21.4 | 29.7 | 0.72 | 10.2 | 40.2 | 20.00 | 21.1 | 30.1 | 0.70 |
|  |  | 10.0 | 39.4 | 20.03 | 22.6 | 29.6 | 0.76 | 9.9 | 39.3 | 20.00 | 21.4 | 29.1 | 0.73 |
|  | 70 | 10.2 | 68.5 | 19.93 | 23.3 | 30.1 | 0.77 | 10.3 | 67.1 | 19.95 | 23.2 | 30.3 | 0.77 |
|  |  | 10.1 | 67.3 | 20.03 | 23.1 | 29.8 | 0.77 | 10.0 | 67.1 | 19.98 | 23.0 | 29.5 | 0.78 |
| 20 | 10 | 20.0 | 12.1 | 20.05 | 41.3 | 59.2 | 0.70 | 19.9 | 12.1 | 20.02 | 26.4 | 59.0 | 0.45 |
|  |  | 19.8 | 11.2 | 19.93 | 40.8 | 58.5 | 0.70 | 19.7 | 12.0 | 19.98 | 27.6 | 58.3 | 0.47 |
|  | 40 | 19.9 | 39.7 | 20.00 | 42.5 | 58.7 | 0.72 | 20.0 | 39.8 | 19.98 | 30.6 | 59.0 | 0.52 |
|  |  | 19.3 | 40.7 | 19.98 | 41.6 | 57.0 | 0.73 | 19.7 | 39.8 | 20.00 | 30.3 | 58.3 | 0.52 |
|  | 70 | 19.8 | 67.7 | 19.98 | 38.4 | 58.6 | 0.66 | 19.8 | 68.5 | 20.00 | 35.2 | 58.5 | 0.60 |
|  |  | 19.8 | 68.9 | 20.00 | 37.9 | 58.7 | 0.65 | 19.9 | 68.5 | 19.97 | 35.0 | 58.9 | 0.59 |
| 30 | 10 | 29.5 | 12.3 | 20.00 | 59.6 | 87.2 | 0.68 | 29.8 | 12.9 | 19.98 | 17.1 | 88.1 | 0.19 |
|  |  | 29.4 | 12.0 | 20.02 | 58.8 | 87.1 | 0.67 | 29.7 | 13.1 | 20.02 | 22.0 | 88.0 | 0.25 |
|  | 40 | 29.5 | 40.3 | 19.98 | 58.5 | 87.1 | 0.67 | 29.6 | 41.5 | 20.02 | 26.7 | 87.7 | 0.30 |
|  |  | 29.2 | 40.4 | 20.03 | 60.9 | 86.4 | 0.71 | 28.6 | 40.4 | 19.98 | 27.8 | 84.5 | 0.33 |
|  | 70 | 29.8 | 69.5 | 19.98 | 58.4 | 88.0 | 0.66 | 29.3 | 69.6 | 20.00 | 38.4 | 86.6 | 0.44 |
|  |  | 29.7 | 70.1 | 20.00 | 55.9 | 87.8 | 0.64 | 29.6 | 69.2 | 20.00 | 35.5 | 87.4 | 0.41 |
| 40 | 10 | 39.0 | 13.0 | 19.98 | 75.0 | 115.2 | 0.65 | 39.0 | 13.4 | 20.02 | 9.3 | 115.6 | 0.08 |
|  |  | 38.9 | 13.0 | 19.95 | 78.5 | 114.8 | 0.68 | 38.9 | 13.4 | 20.00 | 12.1 | 115.1 | 0.11 |
|  | 40 | 39.2 | 41.0 | 19.98 | 78.5 | 115.9 | 0.68 | 39.4 | 41.1 | 20.00 | 16.1 | 116.6 | 0.14 |
|  |  | 38.6 | 40.9 | 20.02 | 78.8 | 114.4 | 0.69 | 39.1 | 40.9 | 19.98 | 17.4 | 115.6 | 0.15 |
|  | 70 | 38.9 | 70.9 | 20.03 | 72.9 | 115.1 | 0.63 | 39.3 | 69.6 | 19.98 | 23.7 | 116.1 | 0.20 |
|  |  | 38.8 | 71.0 | 20.00 | 71.8 | 114.9 | 0.63 | 38.5 | 70.4 | 20.02 | 25.8 | 113.9 | 0.23 |

## Additional statistical results on Table S3 data; second category, [^11^C]CO_2_ and [^11^C]CH_4_ yields

**Effect of nominal current and temperature on the second category measured radioactivities of [^11^C]CO_2_ and [^11^C]CH_4_**

**[^11^C]CO_2_**

Tukey’s *post hoc* test. Detailed differences across the simple main effect of temperature was examined after multiple comparison, which demonstrated significantly higher radioactivity at 40 °C compared to 70 °C, when the nominal current was 20 µA (P = 0.0120). In addition at 40 µA, significantly higher A_EOB_(^11^CO_2_) was observed at 10 °C (P = 0.0055) and 40 °C (P = 0.0003) compared to 70 °C.

Furthermore, the simple main effect of nominal current was examined, which showed significantly higher A_EOB_(^11^CO_2_) at 40 µA compared to lower nominal current values regardless of the temperature (P < 0.0001).

**[^11^C]CH_4_**

Tukey’s *post hoc* test. Detailed differences across the simple main effect of *T* was examined after multiple comparison, which demonstrated significantly higher radioactivity at 70 °C compared to 10 °C, when the nominal current was 10 µA (P = 0.0115). At 20 µA, 30 µA, and 40 µA, significantly higher A_EOB_(^11^CH_4_) was observed at 70 °C compared to 10 °C (ranging from P < 0.0001 to P = 0.0003) and 40 °C (ranging from P < 0.0001 to P = 0.0188), which confirmed that the highest measured radioactivities were gained at higher temperature. Interestingly, the highest radioactivity was measured at 30 µA, while the measured A_EOB_(^11^CH_4_) at 40 µA could not exceed any A_EOB_(^11^CH_4_) at the corresponding *T* setting in other *I* groups.

Furthermore, the simple main effect of nominal current was examined, which showed significantly higher A_EOB_(^11^CH_4_) at 20 µA compared to 10 µA (P = 0.0002) or 30 µA (P = 0.0012), or 40 µA (P < 0.0001) when the temperature was 10 °C. At 40 °C, significantly higher A_EOB_(^11^CH_4_) was also observed at 20 µA compared to 10 µA (P = 0.0002) or 40 µA (P < 0.0001), which was similar with the temperature of 70 °C (P < 0.0001).

# **Table S4**. Measured data from the third category.

Collected radioactivities, A_EOB_(^11^CH_4_), are decay corrected to EOB. *I* is the irradiation current, measured from the target grid and body. To get the average irradiation current on the target gas, *I* is corrected by the grid factor. Theoretical A_EOB_(^11^C) is calculated based on the grid corrected irradiation values. *T* is the measured target chamber temperature, and *t* is the recorded irradiation time.

| **3^rd^ cat.** | **[^11^C]CH_4_** | | | | | | | |
| --- | --- | --- | --- | --- | --- | --- | --- | --- |
|  |  | Fixed | Varied | Nominal | Delivered | Meas. | Theor. |  |
| ***Nominal  current*** | ***I*** | ***T*** | ***t*** | ***Charge*** | ***Charge*** | ***A*_EOB_(^11^CH_4_)** | ***A_EOB_*(^11^C)** | ***Meas./theor.*** |
| [μA] | [μA] | [°C] | [min] | [µA∙min] | [µA∙min] | [GBq] | [GBq] |  |
| 10 | 10.0 | 39.2 | 11.67 | 120 | 117 | 14.0 | 19.6 | 0.72 |
|  | 10.2 | 39.1 | 22.72 | 240 | 231 | 21.9 | 32.8 | 0.67 |
|  | 10.1 | 39.1 | 34.92 | 360 | 353 | 29.2 | 42.1 | 0.69 |
|  | 10.1 | 39.2 | 45.92 | 480 | 461 | 31.8 | 47.6 | 0.67 |
|  | 10.3 | 39.1 | 56.03 | 600 | 576 | 34.1 | 52.5 | 0.65 |
|  | 10.1 | 39.1 | 75.62 | 800 | 766 | 37.5 | 56.1 | 0.67 |
| 20 | 20.1 | 40.3 | 5.92 | 120 | 119 | 13.1 | 22.0 | 0.59 |
|  | 19.9 | 39.8 | 11.90 | 240 | 236 | 22.6 | 39.6 | 0.57 |
|  | 20.0 | 39.7 | 17.67 | 360 | 354 | 32.1 | 54.2 | 0.59 |
|  | 19.9 | 39.6 | 23.75 | 480 | 473 | 34.7 | 66.1 | 0.52 |
|  | 20.0 | 39.6 | 29.50 | 600 | 591 | 39.8 | 76.0 | 0.52 |
|  | 20.0 | 39.6 | 39.33 | 800 | 785 | 42.0 | 88.2 | 0.48 |
| 40 | 39.6 | 40.3 | 3.05 | 120 | 121 | 5.7 | 23.4 | 0.25 |
|  | 39.7 | 40.7 | 6.08 | 240 | 241 | 8.6 | 44.5 | 0.19 |
|  | 39.9 | 40.8 | 9.02 | 360 | 360 | 11.0 | 63.2 | 0.17 |
|  | 40.0 | 40.9 | 12.00 | 480 | 480 | 12.1 | 80.4 | 0.15 |
|  | 39.7 | 40.9 | 15.10 | 600 | 600 | 16.0 | 95.7 | 0.17 |
|  | 39.9 | 40.8 | 20.03 | 800 | 799 | 18.0 | 118.2 | 0.15 |

# **Table S5**. Means and standard deviations (SD) of saturation activities Y_SAT_ [GBq/µA] at temperatures 10 °C, 40 °C and 70 °C.

[^11^C]CO_2_ and [^11^C]CH_4_ productions at various nominal irradiation currents (compare to data groups at Fig. 2).

| Nominal | **[^11^C]CO_2_ production** | | **[^11^C]CH_4_ production** | |
| --- | --- | --- | --- | --- |
| current | **Y_SAT_ [GBq/µA]** | | **Y_SAT_ [GBq/µA]** | |
| µA | Mean | SD | Mean | SD |
| 10 | 5.88 | 0.24 | 5.52 | 0.61 |
| 20 | 5.46 | 0.27 | 4.15 | 0.49 |
| 30 | 5.31 | 0.18 | 2.54 | 0.74 |
| 40 | 5.21 | 0.21 | 1.19 | 0.45 |
| Pooled mean ± SD: | 5.47 ± 0.30 |  |  |  |
